# Procalcitonin: a promising diagnostic marker for sepsis and antibiotic therapy

**DOI:** 10.1186/s40560-017-0246-8

**Published:** 2017-08-03

**Authors:** Ashitha L. Vijayan, Vanimaya, Shilpa Ravindran, R. Saikant, S. Lakshmi, R. Kartik, Manoj. G

**Affiliations:** Diagnostic Products Division, Corporate R&D Centre, HLL Lifecare Limited, Akkulam, Sreekariyam (P.O), Trivandrum, Kerala India

**Keywords:** Procalcitonin, Sepsis, Antibiotic therapy, Diagnostic marker

## Abstract

**Background:**

Sepsis is a global healthcare problem, characterized by whole body inflammation in response to microbial infection, which leads to organ dysfunction. It is becoming a frequent complication in hospitalized patients. Early and differential diagnosis of sepsis is needed critically to avoid unnecessary usage of antimicrobial agents and for proper antibiotic treatments through the screening of biomarkers that sustains with diagnostic significance.

**Main body of abstract:**

Current targeting conventional markers (C-reactive protein, white blood cell, tumour necrosis factor-α, interleukins, etc.) are non-specific for diagnosing sepsis. Procalcitonin (PCT), a member of the calcitonin super family could be a critical tool for the diagnosis of sepsis. But to distinguish between bacterial versus viral infections, procalcitonin alone may not be effective. Rapid elevation in the concentration of procalcitonin and other newly emerging biomarkers during an infection and its correlation with severity of illness makes it an ideal biomarker for bacterial infection. Beside this, the procalcitonin levels can be used for monitoring response to antimicrobial therapy, diagnosis of secondary inflammations, diagnosis of renal involvement in paediatric urinary tract infection, etc.

The present article summarizes the relevance of procalcitonin in the diagnosis of sepsis and how it can be useful in determining the therapeutic approaches.

**Conclusion:**

Further studies are needed to better understand the application of PCT in the diagnosis of sepsis, differentiating between microbial and non-microbial infection cases and determining the therapeutic approaches for sepsis.

## Background

During the course of evolution, our immune system has eventually developed to deal with infectious pathogen invasions by various host defence mechanisms. Inflammatory response is one of the primary responses to a microbial invasion, [1] which leads to the systemic illness which is referred to as sepsis. Its severity correlates with mortality [2–5]. It may occur as a result of infections acquired from community, hospitals or other healthcare facilities. There is an alarming number of 18 million new sepsis cases reported each year worldwide with mortality rate ranging from 30–50% [6]. Intensive care case pattern study reported frequent prevalence of sepsis in India, with 28.3% of patients contact sepsis during ICU stay and have 34% mortality rate [7].

All types of microbes like bacteria, virus, fungi and parasites can cause sepsis, but bacteria cause the most common pathogenic invasion [8–10]. During sepsis, the microorganisms invade to the blood stream and directly proliferate locally and release various virulent factors into the bloodstream [11]. These products can stimulate the release of endogenous mediators of sepsis from endothelial cells, monocytes, macrophages neutrophils and plasma cell precursors [12]. Sepsis-related inflammatory response arise when the body attempts to neutralize pathogenic infection which in turn leads to the activation of various mechanism with the immune cells to secrete inflammatory protein which in turn damage tissues and organs of the host [13, 14]. Clinical symptoms of sepsis include tachycardia, tachypnea, fever, leucocytosis, etc. Usually severe sepsis is accompanied with hypoperfusion or dysfunction of at least one organ. Sepsis associated with multiple organ dysfunction syndrome (MODS) or hypotension is known as septic shock [15].

Early diagnosis and prompt antimicrobial therapy is crucial in the treatment of sepsis for saving lives. Sepsis is a systemic inflammatory response syndrome (SIRS) that affect all organs. Hence, host responses including cytokine, cell markers, receptor biomarkers, coagulations, vascular endothelial damage, vasodilation, organ failure and scientific advancement in the field of molecular biology can equip us to screen wide range of protein markers in acute phase of sepsis development that helps in identifying relevant biomarkers to diagnose sepsis [16].WBC, C-reactive protein (CRP) and interleukin-1 (IL-1) are the conventional markers used for diagnosis of sepsis. Compared to CRP, PCT has better diagnostic and prognostic value and will clearly distinguish viral and bacterial meningitis [17]. Cytokines like TNF-α, IL-1 and IL-6 are elevated during sepsis, but they do not possess sufficient sensitivity or specificity for the development of clinical markers [18]. Blood culture is considered as the gold standard for the confirmation of bacteraemia which can isolate and identify the causative agent and subsequently test the antimicrobial sensitivity, but the delayed process of bacterial culture emphasises the early diagnosis of sepsis [19]. Several studies mentioned the advantages of the precursor molecule of calcitonin, namely procalcitonin as a biomarker for sepsis. The serum PCT level rises rapidly than CRP levels and peaks within very short time; moreover, if the patient responds appropriately to the treatment, the level of PCT returns to normal range faster than CRP which makes it a better biomarker for sepsis [20]. In general, PCT alone or in combination with other biomarkers would serve as a promising tool for understanding the prediction, cause, diagnosis, progression, regression and outcome of the treatment regimes.

## History of procalcitonin

**Fig. 1:**
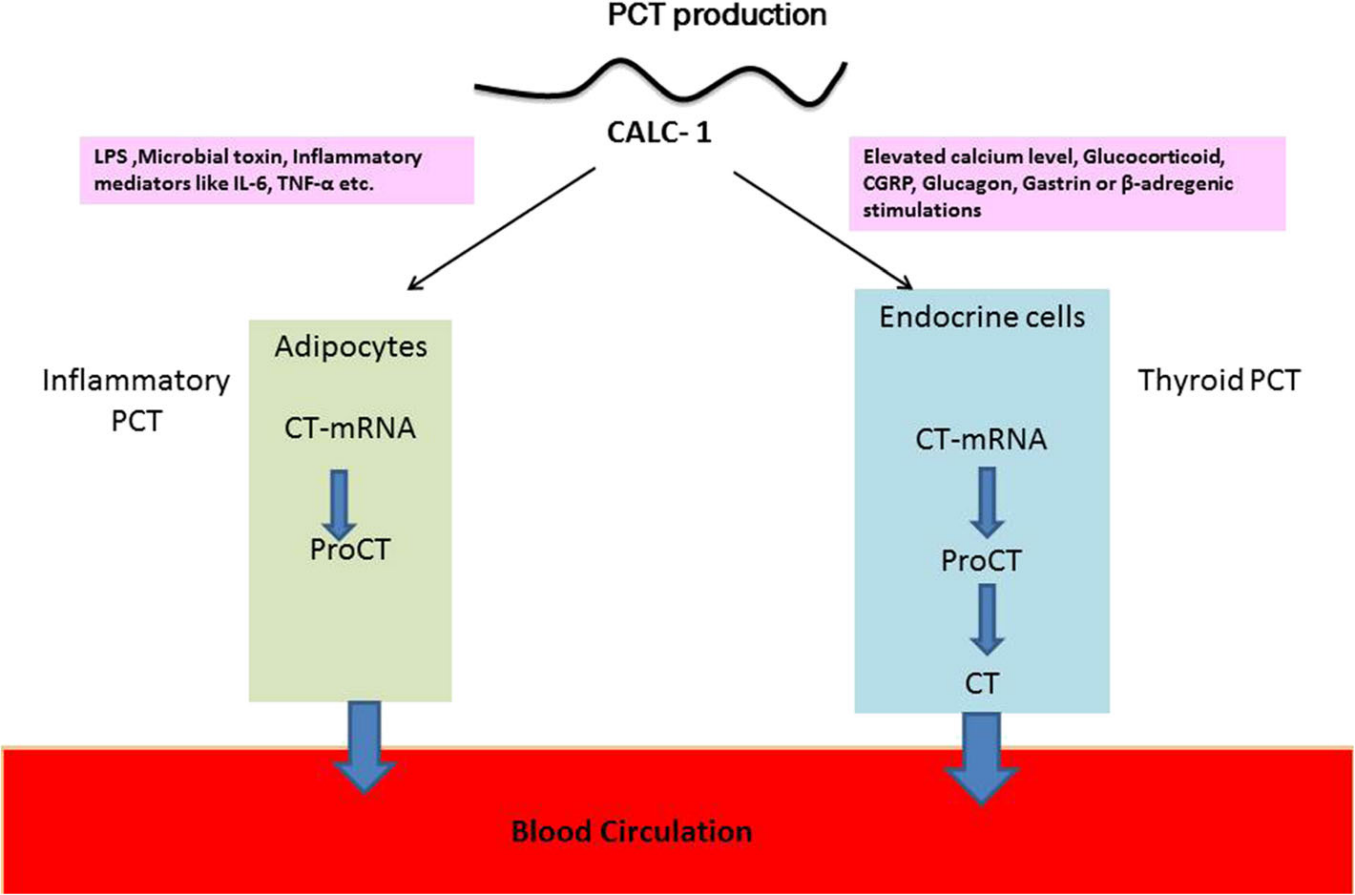
Fate of procalcitonin during inflammation and normal condition

In 1975, Moya F et al. suggested the existence of a precursor for calcitonin in chicken. The large biosynthetic molecule splits intracellularly to generate the hormone, and they named it as procalcitonin [21]. Allison’s study (1981) in RNA isolated from human medullary carcinoma demonstrated the synthesis of calcitonin as a precursor protein molecule in human [22]. Later studies show that calcitonin is secreted after a sequential Co and post translational modification like glycosylation protiolytic cleavage, etc. [23]. In healthy individuals, PCT is produced in thyroid C cells, from a CALC-1 gene located on chromosome 11. The mRNA product is known as preprocalcitonin. It is further modified to 116 amino acid procalcitonin. Finally, it is cleaved into 3 distinct molecules; active calcitonin (32 amino acid), katacalcitonin (21 amino acid) and N-terminal procalcitonin (57 amino acid). Calcitonin hormone is involved in the homeostasis of calcium and phosphorous [24]. Normally, CALC-1 gene in thyroid C cells are induced by elevated calcium level, glucocorticoid, calcitonin gene-related peptide (CGRP), glucagon, gastrin or β-adregenic stimulations. Practically, all the PCT formed in thyroid C cells are converted to calcitonin so that no PCT is released into the circulation. Hence, the PCT level in healthy subjects is very low (0.05 ng/mL) but the inflammatory release of PCT is independent of the above regulations. During inflammation, PCT is produced mainly by two alternative mechanisms; direct pathway induced by lipopolysaccharide (LPS) or other toxic metabolite from microbes and indirect pathway induced by various inflammatory mediators like IL-6, TNF-α, etc. (Fig. 1).

In bacterial septicaemia, PCT is produced by alternate pathways, either directly or indirectly. For better understanding of the pathophysiology of calcitonin precursor in sepsis, an experiment was conducted in animal (hamsters) analogue to human sepsis [25]. During sepsis, ubiquitous and uniform expressions of calcitonin (CT) mRNA in multiple tissues were observed in hamsters. In healthy hamsters, PCT mRNA was isolated primarily from the thyroid with minute amounts of synthesis associated with lung tissue. The next set of hamsters was infected with gram-negative bacteria, and the level of PCT mRNA in various tissues and cells were observed. White blood cells (WBC), spleen, kidney, adipocytes, pancreas, colon and brain showed a significantly elevated level of PCT mRNA. Many reports are available which shows that PCT levels are elevating rapidly between 2 and 6 h which peaks within 6–24 h during bacterial infection.

## Procalcitonin as diagnostic tool

An ideal biomarker should possess high diagnostic accuracy, for an early and rapid diagnosis. PCT is a recently re-discovered biomarker that fulfils many of these requirements especially in comparison to conventional and widely used other biomarkers that have demonstrated superior diagnostic accuracy for a variety of infections, including sepsis. PCT is helpful for early detection of sepsis as well as to monitor the antimicrobial treatment regimen. In fact, PCT can be a useful tool for antimicrobial stewardship and its utilization may safely lead to significant reduction of unnecessary administration of antimicrobial therapy. Laboratories and clinicians must comprehend the precincts of the present microbiological methods and the need for highly sensitive biomarker assays to facilitate accurate diagnosis and goal directed therapy in patients suspected of sepsis.

During sepsis conditions, microbes and their antigens stimulate numerous anti-inflammatory mediators, which will trigger the host immune response. Precursors, mature forms and degradation products of these mediators penetrate from the site of action into the circulation, where which can be measured theoretically. These substances can be measured as surrogate markers for the diagnosis and the severity of infection. Exalted production of PCT during bacterial and its association with sepsis was first demonstrated by Asscot et al. [26]. The actual mechanism of production of PCT during infection is not known, but it assumes that bacterial lipopolysaccharides and sepsis released cytokines modulate the liver and peripheral blood mononuclear cells to produce PCT. Microbial infection induces the elevated expression of CALC 1 gene followed by the release of PCT product which is correlated with severity of disease and mortality.

The PCT as a biomarker proved successfully its clinical usefulness in determining the presence of sepsis. Moreover, it has been shown to correlate the extent and the severity of microbial invasion. It clearly showed the significance of early diagnosis of bacterial infected sepsis. PCT can be used for early detection of sepsis and prediction of outcome after major trauma. Muller et al. conducted a study in patients with community-acquired pneumonia; the serum PCT concentration could differentiate bacterial from viral causes. Out of 545 patients with pneumonia symptoms consulting at the emergency department (ED), 373 were suspected with true bacterial pneumonia, with an area under the receiver operating characteristics (AUROC) curve for the PCT to predict bacterial pneumonia of 0.88. CRP was slightly less efficient (AUROC = 0.76) than PCT. PCT ˃0.1 ng/ml predicted bacterial pneumonia with 90% sensitivity and 59% specificity, whereas PCT ˃1 ng/ml showing 43% sensitivity and 96% specificity [27]. Several meta-analysis data suggest of heterogeneity for the PCT testing; however, elevated PCT concentrations strongly associated with all-cause mortality in sepsis patients [28].

Muller and colleagues conducted a study in consecutive critically ill patients to compare the usefulness of serum concentration of calcitonin precursor, CRP, IL-6 and lactate for the diagnosis of sepsis. Blood samples were collected at variable intervals during the course of disease (systemic inflammatory response syndrome (SIRS), sepsis and severe sepsis and septic shock. Serum concentration of calcitonin precursor, CRP, IL-6 and lactate were elevated according to the severity of illness. Based on receiver operating characteristic (ROC) curve analysis, they concluded that PCT is the most reliable marker for the diagnosis of sepsis, with 89% of sensitivity and 94% of specificity [29]. Ibrahim et al. evaluated the utility of PCT as a routine biochemical tool compared to the traditional inflammatory marker CRP. They simultaneously measured PCT and CRP in 73 medico surgical ICU patients; according to the American College of Chest Physician (ACCP) criteria-based study group, 75% cases revealed SIRS in clinical representation. They observed 75% of diagnostic accuracy, 72% of specificity and sensitivity of 76% for PCT and concluded that PCT is superior to CRP in terms of accuracy in identification and assessment of severity of sepsis [30].

Study of Young et al. [31] evaluated the ability of PCT as an early detector of septic shock in patients with acute pyelonephritis secondary to ureteral calculi. They considered 49 patients and divided them into 2 groups: with and without septic shock. Platelet count, PCT, CRP, creatinine, erythrocyte sedimentation rate (ESR), albumin and white blood cells (WBC) were measured at the time of admission to the emergency department before administrating antibiotic treatment. Univariate analysis shows higher PCT and CRP level and higher positive blood culture rate during septic shock. The multivariate model reveals that lower platelet count and higher PCT level are independent risk factors of septic shock. In ROC curve, the AUC (area under curve) was wider for PCT (0.929) compared to platelet count (0.822). At the cut-off of 0.52 ng/ml, PCT shows high sensitivity (86.7%) and specificity (85.3%). The study demonstrated that elevated PCT is an early independent predictor of development of septic shock in patients with sepsis induced by acute pyelonephritis associate with ureteral calculi.

## Comparison of procalcitonin and presepsin/sCD-14

In 2004, presepsin was identified as a diagnosis marker and evaluated for sepsis. It became an alternative biomarker to aid the diagnosis of sepsis [32]. Recent studies shows that soluble cluster of differentiation 14 (sCD14) plays a significant role as biomarker with respect to diagnosis of sepsis. CD14 is a surface marker constituent of glycoprotein on monocytes and macrophages (mCD14) and serves as a high-affinity receptor towards lipopolysaccharides (LPSs), which is an essential building block of outer cell wall of gram-negative bacteria, and LPS-binding proteins (LPBs). CD14 binding to toll-like receptor 4 (TLR4) lead to activation of pro-inflammatory signalling cascade in bacterial infection [33, 34].

Along with PCT, other biomarkers alone or in combination are used in the diagnosis of sepsis, including presepsin, C-reactive protein (CRP), interleukin (IL), etc. However, the clinical value of these biomarkers independently or in combination is still at investigative stages [35]. Presepsin exists in the blood and urine of humans and comprises 99% of the total amount of CD14 in the human body, with a normal concentration of 2–6 μg/ml in serum [36]. When this was compared to PCT, presepsin also showed a similar diagnostic accuracy for sepsis with respect to area under curve (AUC) (Table 1) [37–40]. Accuracy of presepsin was similar to that of PCT, although presepsin had some superiority in the management of patients but Food and Drug Administration (FDA) approved PCT as a more reliable marker for sepsis. PCT level above 2.0 ng/ml on the first day of ICU admission could be associated with a higher risk for progression to severe sepsis and/or septic shock than PCT levels below 0.5 ng/mL. This diagnostic approach is also recommended in various guidelines [41].

**Table 1:** Comparison of diagnostic potential of procalcitonin and presepsin

Reports	No. of subject	Area under curve (AUC) for the diagnosis of sepsis	
		Procalcitonin	Presepsine
Dunja Mihajlovic et al. (2017) [3]	100	0.750	0.730
Christian Leli et al.(2016) [38]	0 92	0.876	0.788
Kada Klouche et al. (2016) [39]	144	0.800	0.750
Enguix-Armada A et al.(2016) [40]	388	0.989	0.948

## Procalcitonin as a tool for antibiotic therapy

The introduction of antibiotics was in mid-20th century. Misuse and overuse of antibiotics launch the next misery known as antibiotic resistance by pathogens. Judicious use of antibiotics is of vital importance in clinical therapy [42]. Scott Fridin et al. analysed the Centres for Disease Control and Prevention (CDC) which conducted Emerging Infections Program (EIP), and thus a national administrative database (Marketscn Hospital Drug Database) was prepared to assess the potential for improvement of inpatient antibiotic prescribing. According to their study, reduction of incorrect antibiotic prescription can improve the use and patient safety [43].

**Fig. 2:**
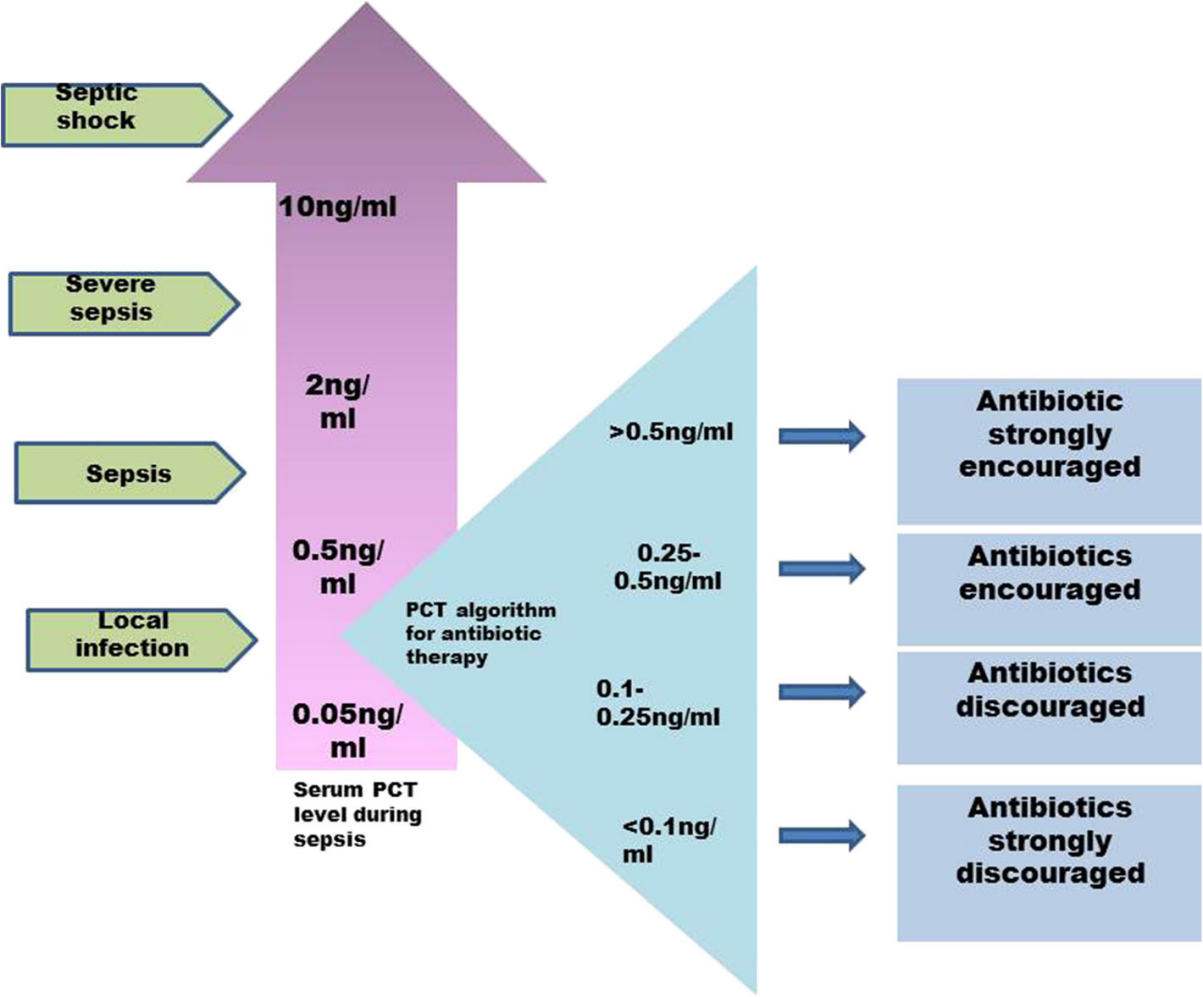
PCT algorithm for antibiotic therapy

Implementation of antibiotic stewardship helps to control unnecessary antibiotic prescribing as well as ensure the efficiency of treatment [44]. Inappropriate usage of medicines may lead to the development of antibiotic resistance in patients [45]. It is obligatory to reduce ‘blind’ prescription of drugs to avoid the evolution of secondary infection to antibiotics and obstruct the occurrence of drug resistance. An ideal marker should assist early diagnosis and capabilities to track the disease and facilitate the therapeutic interventions and decisions. The PCT is a better choice, compared to other markers which satisfy these features. An algorithm based on serial measurement of PCT can reduce the antibiotic exposure in septic patients [46]. According to the level of serum PCT, therapeutic decisions in patients were taken (Fig. 2). Based on reports, in transplant recipients, to minimize delays in the diagnosis of sepsis, it is paramount to recognize the specific risk factors for infection associated with each allograft type. Hence, PCT can be a better biomarker in detecting bacterial sepsis at initial stages. But the major limitations are to identify the causative bacteria. In addition, the particular surgical techniques involved in each type of transplantation may be closely related to the clinical manifestations of the infection process. Hence, further culturing and gram staining is required to identify the type of bacteria for further accurate treatments.

A study conducted in acutely febrile patients reveals that measurement of PCT is helpful in differentiating bacteria and non-bacterimic infectious episodes in patients. Based on the above observations, serum PCT level measurement is recommended for the guidance of antibiotic therapy [47]. Stolz D et al. evaluated the efficacy and safety of PCT guidance compared to standard therapy with antibiotic prescriptions in patients experiencing exacerbations of chronic obstructive pulmonary disease (COPD) [48]. Compared to standard therapy, PCT guidance reduce the antibiotic exposure (relative risk (RR), 0.56; 95% confidence interval (CI), 0.43 to 0.73; *p* < 0.0001) and antibiotic prescription (40 vs. 72%, respectively; *p* < 0.0001).

Schroeder et al. conducted a study in surgical intensive care patients with severe sepsis in which two classes were considered, PCT guided and control. For all patients, drug administration was based on microbiological spectrum. When the clinical signs of infection improved and PCT level decreased to <35% of the initial value, the antibiotic treatment was discontinued in PCT-guided patients. In control group, treatment was based on empirical rules. They observed that PCT-based algorithm reduces the use of antibiotic as well as the expense of treatment [49]. Lavrentieva et al. reported that PCT-guided algorithm for antibiotic therapy may contribute to the reduction of antibiotic exposure in burn intensive care unit. They enrolled 46 burn ICU patients for the study, wherein 24 patients received the therapy based on PCT guidance, which resulted in a smaller antibiotic exposure (10.1 ± 4 vs. 15.3 ± 8 days) without any negative impact on clinical outcome [50].

Kip and colleagues assessed the cost effectiveness of PCT-based algorithm. It was found to reduce the length of hospital stay, number of blood cultures and the duration of antibiotic therapy [51]. Antibiotic treatment based on PCT monitoring is a sensitive way of antibiotic usage in ICU patient with severe sepsis and septic shock [52]. Anna Prkno and co-workers reviewed various studies regarding the clinical trials and compared PCT-guided antibiotic stewardship with standard care and hospital mortality, duration of antimicrobial therapy and length of stay in the ICU. It was noticed that PCT guidance could not make better change in the mortality rate, but there was definitely a noteworthy effect on the duration of antimicrobial treatment [53, 54].

## Future of PCT for the management of sepsis

Various studies are published revealing the wide application of PCT in medical field. Before selecting PCT as a biomarker, its limitations have to be studied. Further studies should be conducted to disclose even more application of PCT. The assay used for the detection must distinguish PCT levels between healthy individuals, non-bacteremia patients and progressing SIRS. More sensitive assays should be developed to take forward the studies to the clinical level [55, 56].

## Conclusion

The PCT is a unique biomarker having wide range of application in the medical field, compared to other conventional markers for sepsis. However, to diagnose invasive bacterial infection and their severity assessment of PCT levels alone may not be enough. Because of the possible complication in diagnosis of sepsis and the challenge in differentiating between microbial and non-microbial infection cases, it is unlikely that a single biomarker serve as an effective diagnosis tool. A combination of biomarkers may be more functional in the case of clinical application, but this may require further investigation in various aspects as a reliable diagnostic tool [57, 58]. Measurement of combinational biomarkers may require reliable and cost effective technology development. Selection of biomarkers plays a crucial role in technology development; consequently, assessment of combination of biomarkers like procalcitonin, sCD14-ST and other new biomarkers can be made use in evaluation of sepsis in all age groups. Combination of emerging new biomarkers with PCT could be used in terms of good clinical judgement based on which antimicrobial therapy may suggested, thus reducing the prescription and duration of antibiotic treatment. Combinational biomarker with PCT-guided antibiotic stewardship could be properly fabricated to develop a safer and affordable strategy for diagnosis of sepsis and its prognosis.
